# Prognostic value of ALDH2 polymorphism for patients with oropharyngeal cancer in a Japanese population

**DOI:** 10.1371/journal.pone.0187992

**Published:** 2017-12-05

**Authors:** Hirotaka Shinomiya, Hitomi Shinomiya, Mie Kubo, Yuki Saito, Masafumi Yoshida, Mizuo Ando, Masanori Teshima, Naoki Otsuki, Naomi Kiyota, Ryohei Sasaki, Ken-ichi Nibu

**Affiliations:** 1 Department of Otolaryngology-Head and Neck Surgery, Kobe University Graduate School of Medicine, Kobe, Japan; 2 Department of Head and Neck Surgery-Otolaryngology, University of Tokyo, Tokyo, Japan; 3 Department of Medical Oncology and Hematology, Kobe University Graduate School of Medicine, Kobe, Japan; 4 Department of Radiation Oncology, Kobe University Graduate School of Medicine, Kobe, Japan; Duke Cancer Institute, UNITED STATES

## Abstract

**Background:**

Half of Japanese possess a polymorphism of aldehyde dehydrogenase 2(ALDH2), while few white individuals possess this mutation. The purpose of this study was to investigate the possibility of ALDH2 polymorphism as a prognostic factor for oropharyngeal cancer (OPC) among Japanese population.

**Methods:**

We analyzed 82 Japanese patients with OPC treated between 2006 and 2011. The median observation period was 50 months. P16-staining and ALDH2 polymorphisms were investigated. To examine the frequencies of second primary pharyngeal and esophageal cancers (SPPEC),37 Japanese patients with OPC treated at Tokyo University Hospital were included for statistical analysis.

**Results:**

Statistically significant differences were noted in OS among sex, age, N classification, and p16 (p = 0.045, 0.024, 0.020, 0.007, respectively). In addition, OS and DSS rates of the patients with heterozygous ALDH2 tended to be worse than those of the patients with homozygous ALDH2 (p = 0.21, 0.086, respectively). Of note, OS and DSS of the patients with p16-negative OPC and heterozygous ALDH2 was significant poorer than those of the patients with p16-positive OPC (p = 0.002, 0.006, respectively), while there was no significant difference in OS and DSS between patients with p16-positive OPC and patients with p16-negative OPC and homozygous ALDH2.

**Conclusions:**

ALDH2 polymorphism might be a promising prognostic factor for Japanese patients with p16-negative OPC.

## Introduction

Smoking and alcohol consumption are well known classical risk factor for head and neck squamous cell carcinoma (HNSCC) including laryngeal, oropharyngeal and hypopharyngeal cancers similar to esophageal cancer [1–3]. As smoking and alcohol consumption have decreased over the past 3 decades in the United States and Northern Europe, the incidence of HNSCC has steadily decreased in nearly all head and neck subsites. However, the incidence of oropharyngeal cancer (OPC) has increased in conjunction with a marked increase in human papillomavirus (HPV)-associated OPC [4]. As a result, HPV-associated OPC is now more predominant than classical OPC related to alcohol and/or smoking consumption.

Unlike traditional OPC, HPV-associated OPC is not associated with the traditional risk factors of tobacco and alcohol use, but has different characteristics [5]. Pathologically, HPV-associated OPC is poorly differentiated and immunohistochemically positive for p16. Typically, HPV-associated OPC features a small primary lesion with multiple lymph node metastases in relatively young patients. Even in the advanced stage, HPV-associated OPC shows favorable response to chemoradiotherapy and good prognosis [6]. These findings are also true for Japanese populations. Regardless of the treatment modality, patients with HPV-associated OPC show favorable prognosis, both the patients treated with up-front surgery and those treated with chemoradiotherapy [7].

However, the prognostic values of the established risk factors tobacco and alcohol consumption showed a distinct difference between the United States and Japan. Ang *et al*. reported that HPV-status and tobacco smoking were prognostic factors for OPC, but did not mention alcohol consumption [8]. In contrast, Sato *et al*. recently reported that HPV status and alcohol consumption but not smoking were prognostic factors for patients with OPC in Japanese population [9]. The most likely reason for this discrepancy is a difference between Japanese and white individuals in terms of the presence or absence of aldehyde dehydrogenase-2 (ALDH2) polymorphism, a key enzyme for the elimination of acetaldehyde [10]. As individuals homozygous for the ALDH2*2, a variant allele of ALDH2 are unable to metabolize acetaldehyde, those possessing heterozygous ALDH2 (ALDH2*1/2*2) have a 6-fold higher blood acetaldehyde concentration after alcohol consumption than those possessing homozygous ALDH2*1/2*1. Evidence suggests that drinkers with the ALDH2*2 have a higher incidence of HNSCC and esophageal cancer [11–13]. About half of all Japanese possess heterozygous ALDH2 (ALDH2*1/2*2) and 5% possess homozygous ALDH2*2/ALDH2*2, while most white individuals possess ALDH2*1/ALDH2*1 [11, 14–15]. Until now, however, there has been no report on the prognostic significance of ALDH2 polymorphism for the survival rate of the patients with OPC. In this paper, to develop a personalized treatment strategy for individual Japanese patients with OPC, we investigated the prognostic value of ALDH2 polymorphism in addition to that of HVP-status and clinical characteristics for OPC patients.

## Materials and methods

### Patients and materials

Between April 2006 and March 2011, 98 patients with OPC were treated at Kobe University Hospital, Japan. Six patients who received palliative treatment due to their poor health conditions or far-advanced disease were excluded from this study, as were 10 patients whose pathological tissue findings, records of alcohol consumption and smoking history, or written informed consent could not be obtained. As a result, 82 patients with OPC were included for further analysis. Medical records were retrospectively reviewed to obtain information regarding patient characteristics including alcohol consumption and smoking history, extent of disease, treatment, adverse events, and oncological results. The age of the patients ranged from 31 to 85 years old with a median age of 63.8 years and the follow-up period ranged from 2 to 90 months (median: 42 months). The TNM staging system was used to classify tumors in accordance with the American Joint Committee on Cancer classification (fourth edition, 2014). Patient distribution based on TNM classification is provided in Table 1. Most patients were treated by CDDP-based concurrent chemoradiotherapy. Salvage surgery including neck dissection and/or radical resection of primary site were also performed if indicated.

**Table 1:** Distribution of 82 patients according to TN classification.

	No. of Patients (%)						
	N0	N1	N2a	N2b	N2c	N3	Total
T1	4	2	0	2	1	0	9 (11.0)
T2	12	8	3	9	3	5	40 (48.8)
T3	8	0	0	6	3	0	17 (20.7)
T4a	3	3	0	2	5	0	13 (15.9)
T4b	0	0	0	1	1	1	3 (3.7)
Total	27 (32.9)	13 (15.8)	3 (3.7)	20 (24.4)	13 (15.9)	6 (7.3)	82 (100)

### Genotyping

DNA was extracted from paraffin-embedded biopsies or surgical specimens. To determine the genotype of ALDH2, ALDH2 1951G>A (rs671) was subjected to analysis. TaqMan Drug Metabolism Genotyping Assays (Life Technologies Corp., CA, USA) was employed for genotyping using TaqMan SNP Genotyping Mix (ID: C-11703892-10; Life Technologies) according to the manufacturer’s instructions. Results of SNP genotyping were confirmed by PCR assay: f1 (caaattacagggtcaactgc) and r1 (acactcacagttttcacttc) were used to amplify ALDH2*1 allele and f2 (caaattacagggtcaactgc) and r2 (acactcacagttttcacttt) was used to amplify ALDH2*2 allele. PCR products were electrophoresed with 3% agarose gel and photographed. The 133 bp band shows both ALDH2*1 and ALDH2*2. All the results of the PCR assay were consistent with those of the Taqman probe assay. To further enhance the control of genotyping, the sequences of ALDH2 gene were examined by using sequencer for 5 samples. The results were again consistent with those of the TaqMan probe assay.

### P16 immunohistochemistry

To investigate the association of HPV with OPC, we assessed the status of p16 as a surrogate marker of HPV. Immunohistochemical staining for p16 was performed on 4-μm sections cut from formalin-fixed, paraffin-embedded tissue blocks of biopsies or surgical specimens. The slides were deparaffinized and rehydrated through immersion in xylenes and ethanol series. Mouse anti-Human p16^INK4a^ anti-body (CINtec^®^ p16 Histology Kit, Roche Applied Science, Mannheim, Germany) was used for immunohistochemical staining according to the manufacture’s protocol. Positive p16 expression was defined as the presence of strong and diffuse nuclear and cytoplasmic staining in 70% or more of the tumor cells as described elsewhere [16].

### Statistical analysis

Potential correlations between p16 status and clinical features were tested using Fisher’s exact probability test. Kaplan-Meier plots were used to summarize time to event measured from the start of the first treatment. Overall survival rates (OS) and disease specific survival (DSS) rates were compared using the log-rank test. A Cox proportional hazards model was used to determine the relationship between OS, DSS, and other variables. "R" (ver. 3.0.2 2013; R Foundation for Statistical Computing, Vienna Austria) was used for all statistical analyses. A P value less than 0.05 was determined as significant. All the procedures in this study were approved by the Institutional Review Board of Kobe University Hospital (No.1586) and Tokyo University Hospital (No. XXXX). Written informed consent was obtained from all the participants.

## Results

### P16 status and clinical features

Clinical characteristics and p16 expression of 82 patients with OPSCC are summarized in Table 2 (S1 Table). Positive staining for p16 was more common for women than for men (68.7% *vs*. 36.4%; p = 0.025) and more common for non-drinkers than for drinkers (75.0% *vs*. 32.2%; P = 0.004). There was no significant correlation between p16-status and tobacco habit. P-16-positive patients tended to show relatively advanced lymph node (p = 0.054).

**Table 2:** Clinical characteristics of 82 patients.

		No. of patients (%)		P value
		P16-positive	P16-negative	
Sex	Male	24 (69)	42 (89)	0.025
	Female	11 (31)	5 (11)	
Age	Median (range)	60 (46–83)	64 (54–83)	0.021
Primary sites	Base of tongue	9 (26)	11 (23)	0.20
	Tonsil	23 (66)	23 (49)	
	Soft palate	2(6)	9 (19)	
	Posterior wall	1(3)	4 (9)	
T	T1	3 (9)	6 (13)	0.87
	T2	18 (51)	21 (45)	
	T3	8 (23)	9 (19)	
	T4	6 (17)	10 (21)	
N	N0	7 (20)	21 (45)	0.054
	N1	7 (20)	4 (9)	
	N2a	2 (6)	1 (2)	
	N2b	12 (34)	8 (17)	
	N2c	6 (17)	7 (15)	
	N3	1 (3)	5 (11)	
Clinical stage	I	0 (0)	4 (9)	0.23
	II	4 (11)	9 (19)	
	III	8 (23)	8 (17)	
	IV	23 (66)	25 (53)	
Smoking	10 pack-years ≥	15 (43)	12 (26)	0.15
	10 pack-years <	20 (57)	35 (74)	
Alcohol	Nondrinkers	15 (43)	5 (11)	0.004
	Drinkers	20 (57)	42 (89)	

10 packs-years: 1 pack per day for 10 years; Drinkers: patients who have been consuming alcohol daily for ≧ 15years

### Survival rates according to p16-status and N classification

**Fig 1:**
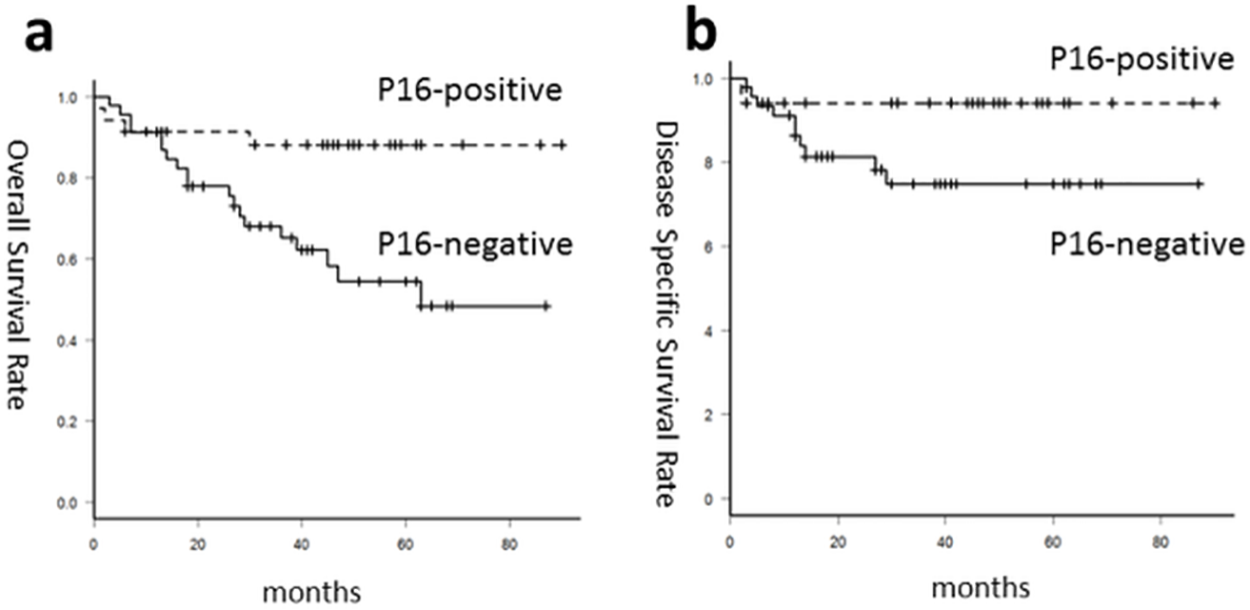
Survival rates according to HPV-status. Survival curve with Kaplan-Meier estimates of (a) overall survival and (b) disease specific survival for patients with HPV-positive (dotted line) and HPV-negative OPC (solid line).

Overall survival was analyzed for 82 patients with OPC. The 3-year OS rate was 87% for patients with p16-positive and 68% for those with p16-negative OPC (p = 0.007), while the corresponding 3-year DSS rates were 87% and 72% respectively (p = 0.057; Fig 1). Among patients with p16-negative OPC, the percentage of patients with heterozygous ALDH2 was relatively higher in comparison with patients with p16-positive OPC (p = 0.21) (Table 3).

**Table 3:** Distribution of patients according to p16-status and polymorphism of ALDH2.

	No. of Patients (%)			
	ALDH2*1/2*1	ALDH2*1/2*2	ALDH2*2/2*2	Total
P16-positive	20 (57)	10 (29)	5 (14)	35
P16-negative	22 (47)	22 (47)	3 (6)	47
Total	42	32	8	82

**Fig 2:**
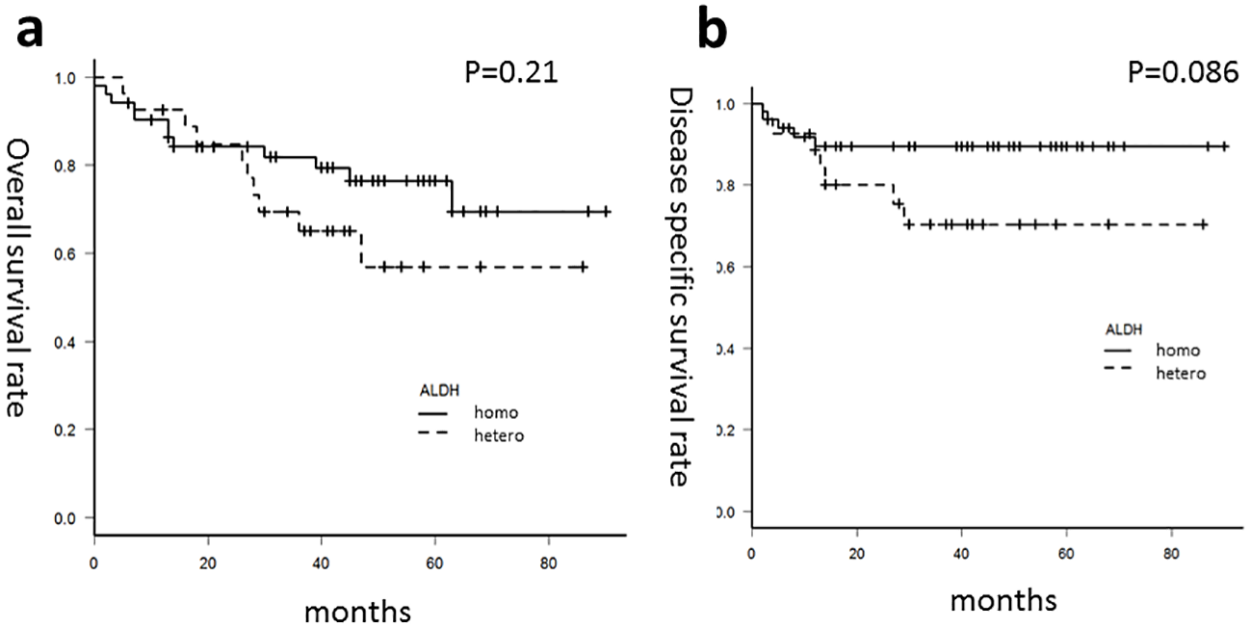
Survival rate according to ALDH2 polymorphism. Survival curve with Kaplan-Meier estimates of (a) overall survival and (b) disease-specific survival for patients with homozygous (solid line) and with heterozygous ALDH2 (dotted line).

First, we evaluated the OS rates of the patients with OPC according to ALDH2 genotype. Although there was no significant difference in the survival rate according to ALDH2 genotype as such, 3-year OS and DSS rates of the patients with homozygous ALDH2 (ALDH2*1/2*1 or ALDH2*2/2*2) tended to be better than those of patients with heterozygous ALDH2 (ALDH2*1/2*2) (p = 0.21 and 0.086, respectively: Fig 2a and 2b). Univariate and multivariate analysis for OS and DSS were summarized in Tables 4 and 5. Statistically significant differences were noted in OS among sex, age, N classification, and p16. (p = 0.045, 0.024, 0.020, 0.007, respectively), but no significant differences were noted in OS among smoking, drinking, ALDH2. Multivariate Cox proportional hazards model analysis identified p16 as an independent prognostic marker (p = 0.047 Table 4).

**Table 4:** Univariate analysis of the risk of death.

Variables		No. of patients	3yOS	P value	3yDSS	P value
**Sex**	**Male**	**66**	**70**	**0.045**	**78**	**0.070**
	**Female**	**16**	**100**		**100**	
**Age**	**60≥**	**28**	**88**	**0.024**	**90**	**0.20**
	**60<**	**53**	**72**		**78**	
**T**	**T1, 2,3**	**66**	**84**	**0.13**	**87**	**0.17**
	**T4**	**16**	**71**		**72**	
**N**	**N0-2a**	**42**	**68**	**0.020**	**81**	**0.89**
	**N2b-3**	**40**	**83**		**83**	
**Smoking**	**10pack-year≥**	**27**	**82**	**0.92**	**86**	**0.75**
	**10pack-year<**	**55**	**72**		**82**	
**Drinking**	**Non-drinker**	**20**	**86**	**0.62**	**86**	**0.99**
	**Drinker**	**62**	**76**		**79**	
**P16**	**Positive**	**35**	**87**	**0.0070**	**93**	**0.050**
	**Negative**	**47**	**68**		**75**	
**ALDH2**	**Homo**	**50**	**82**	**0.21**	**89**	**0.079**
	**Hetero**	**32**	**69**		**70**	

**Table 5:** Multivariate analysis of the risk of death.

Variables		OS			DSS		
		HR	95%CI	P value	HR	95%CI	P value
**Sex**	**Male vs. Female**	**5.3**	**0.70–39**	**0.10**	**-**	**-**	**0.99**
**Age**	**60< vs. 60≥**	**3.4**	**0.99–11**	**0.051**	**-**	**-**	**-**
**N**	**N2b-3 vs. N0-2a**	**0.40**	**0.11–1.02**	**0.054**	**-**	**-**	**-**
**P16**	**Positive vs. Negative**	**0.34**	**0.16–0.98**	**0.047**	**0.30**	**0.067–1.4**	**0.13**
**ALDH2**	**Hetero vs. Homo**	**-**	**-**	**-**	**2.4**	**0.66–6.5**	**0.21**

HR: hazard ratio; CI confidence interval.

**Fig 3:**
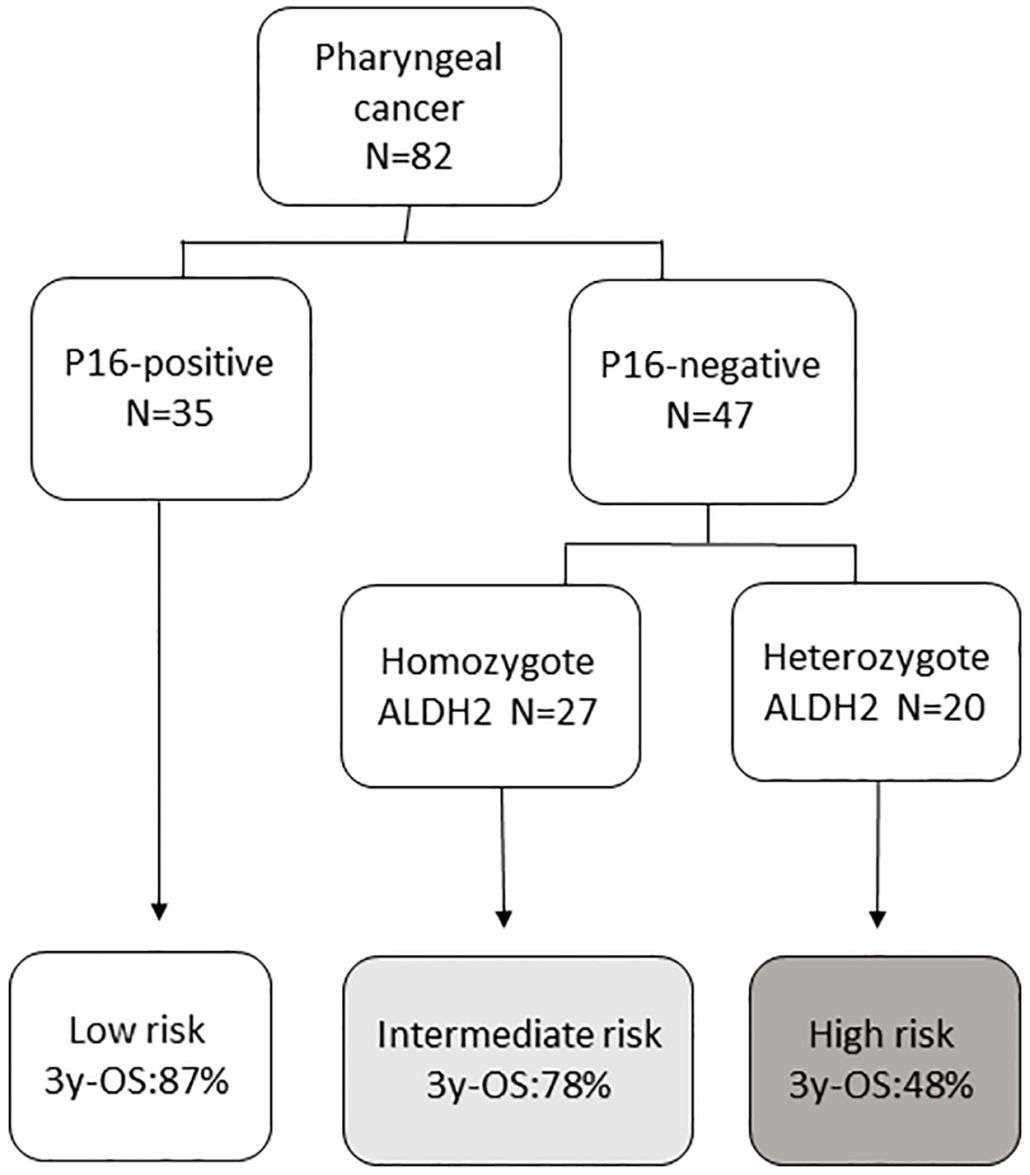
Risk-of-Death Categories according to p16-status and ALDH2 polymorphism. Patients were divided into 3 risk-of-death categories according to p16-status and ALDH2 polymorphism.

**Fig 4:**
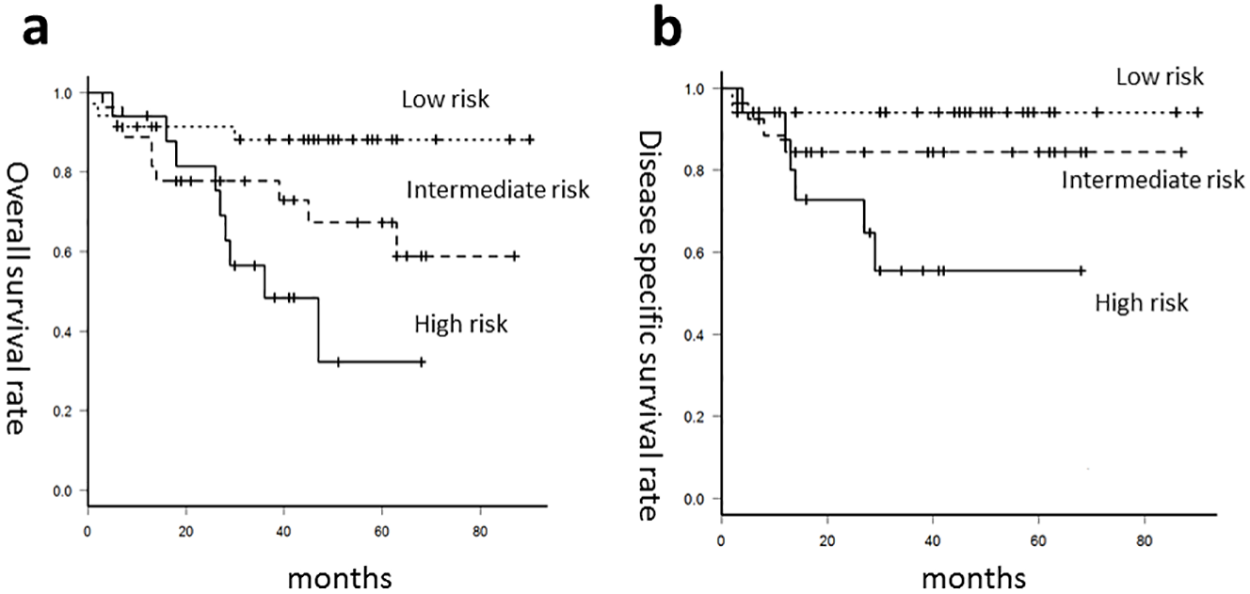
Survival rates according to three risk-of-death categories. Survival curves based on Kaplan-Meier estimates of (a) overall survival and (b) disease-specific survival for patients of the low-risk (small dotted line), the intermediate-risk (large dotted line) and the high-risk group (solid line).

Next, we divided the patients into the following 3 risk-of-death categories: patients with p16-positive tumors (low-risk group), patients with p16-negative tumors and homozygous ALDH2 (intermediate-risk group), and patients with p16-negative tumors and heterozygous ALDH2 (high-risk group) (Fig 3), for evaluation of the OS and DSS rates of these three groups. The 3-year OS rates were 87% for low-risk, 78% for the intermediate-risk, and 48% for high-risk (Fig 4a). The corresponding 3-year DSS survival rates were 93%, 84% and 55% (Fig 4b). Of note, 3-years OS and DSS of the patients with p16-negative OPC and heterozygous ALDH2 was significant poorer than those of the patients with p16-positive OPC (p = 0.002, 0.006, respectively), while there was no significant difference in 3-years OS and DSS between patients with p16-positive OPC and patients with p16-negative OPC and homozygous ALDH2. In addition, 3-years OS rate of patients with ALDH2 heterozygote tended to be worse than that of patients with ALDH2 homozygote among p16-negative OPC (p = 0.15).

### Risk of second primary pharyngeal and esophageal cancers according to p16-status and ALDH2 genotype

**Fig 5:**
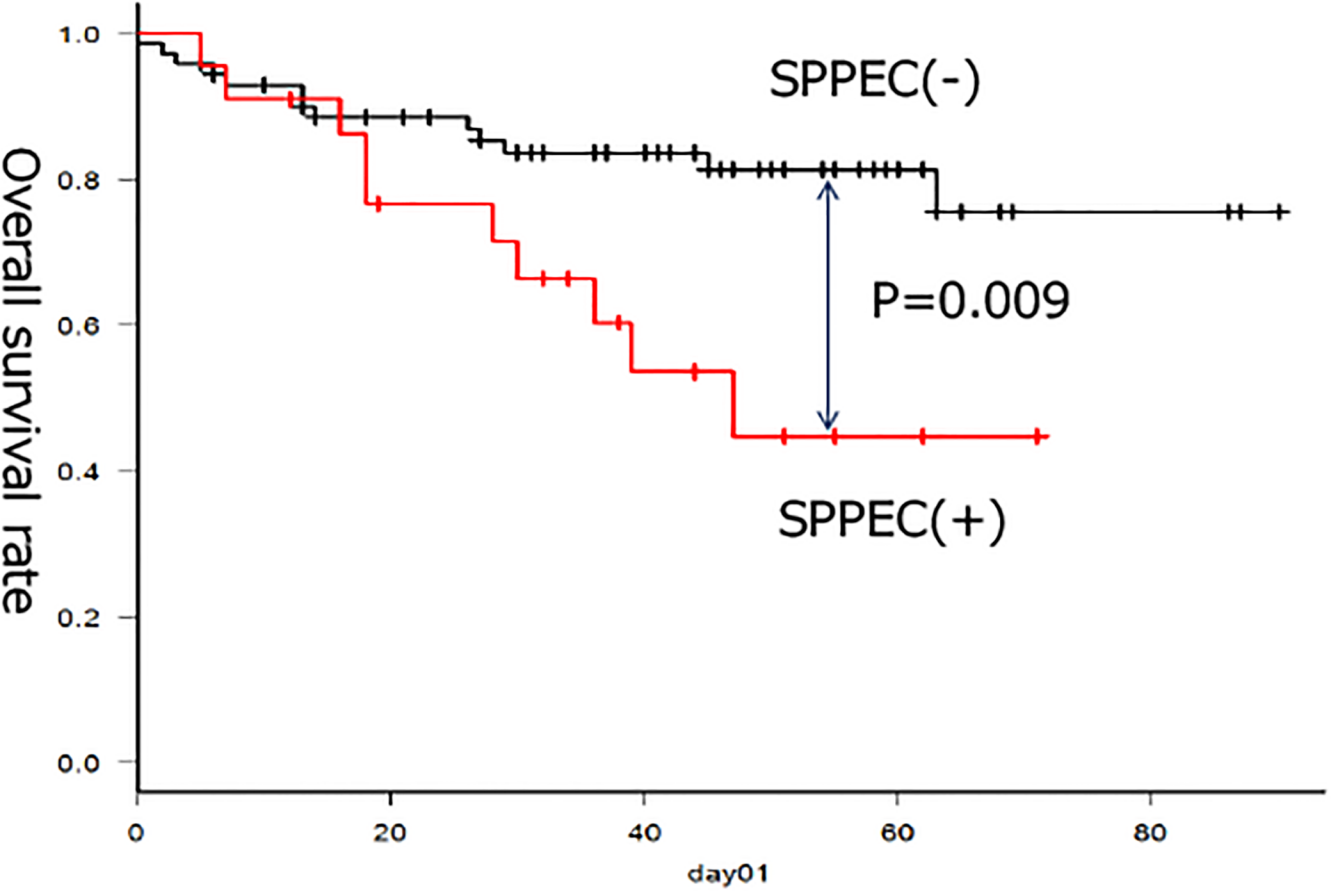
Overall survival rates according to second primary pharyngeal and esophageal cancers. Survival curves based on Kaplan-Meier estimates of overall survival for patients with SPPEC (red line) and without SPPEC (black line).

Since drinkers with heterozygous ALDH2 are at high risk of HNSCC and esophageal cancer, as previous mentioned, we finally investigated the rates of second primary pharyngeal and esophageal cancers (SPPEC) for these patients according to ALDH2 genotype. For this analysis, 37 Japanese patients with OPC treated at Tokyo University Hospital were included for statistical analysis (S2 Table). A total of 29 patients had SPPEC (24%), with SPPEC significantly more common in patients with p16-negative than in those with p16-positive OPC (32% vs 11%, p = 0.017). Moreover among patients with p16-negative OPC, SPPEC occurred more frequently in patients with heterozygous than in those with homozygous ALDH2 (49% vs 18%, p = 0.0065; Table 6). In addition, survival rate of the patients with SPPEC was significantly poorer than those of the patients without SPPEC (p = 0.009; Fig 5).

**Table 6:** Patients with second primary pharyngeal and esophageal cancers according to p16-status and ALDH2 polymorphism.

P16-status	No. of Patients	P value	ALDH2 polymorphism	No. of Patients	P value
P16-positive	5/45 (11%)	0.0017	heterozygote	3/16 (19%)	0.33
			homozygote	2/29 (7%)	
P16-negative	24/74 (32%)		heterozygote	17/35 (49%)	0.0065
			homozygote	7/39 (18%)	

## Discussion

OPC has been on the rise in recent years, especially HPV-associated OPC has dramatically increased not only in the United States and European countries but also in Asian countries [4, 7]. Persistent HPV infection is known to be associated with carcinogenesis of OPC, similar to its association with cervical cancer [17]. Although HPV-associated OPC mostly presents with advanced stages, it reportedly shown favorable response to radiotherapy and chemotherapy as well as surgery. Prognosis of the patients with HPV-associated OPC is significantly favorable in comparison with patients with classical OPC associated with heavy alcohol and smoking consumption, as shown in the present study.

Alcohol consumption and smoking are well recognized as the conventional carcinogenic risk factors for head and neck cancer and esophageal cancer [1–3]. Since about half of all Japanese possess heterozygous ALDH2, which has weak enzyme activity, many studies of the carcinogenic risk of alcohol consumption have been published in the Asian region [1–3, 10–13]. In addition, and in contrast to the USA, as already mentioned, alcohol consumption, rather than tabacco smoking, has been identified as an independent factor for survival of Japanese patients with OPC [9]. These findings led us to the notion that ALDH2 polymorphism might be a possible prognostic factor for OPC in Asian populations.

The present study showed that heterozygous ALDH2 is more common in the patients with p16-negative OPC than in those with p16-positive OPC, suggesting that, in addition to alcohol consumption, heterozygous ALDH2 might also be a carcinogenic factor for patients with p16-negative OPC.

Modifying the previous reports [8–9], we categorized the patients with OPC into three risk-of-death groups according to p16-status and ALDH2 polymorphism to evaluate the significances of ALDH2 genotype for prognosis of the patients with OPC. We found that 3-years OS and DSS of the patients with p16-negative OPC and heterozygous ALDH2 was significant poorer than those of the patients with p16-positive OPC (p = 0.002, 0.006, respectively), while there was no significant difference in 3-years OS and DSS between patients with p16-positive OPC and patients with p16-negative OPC and homozygous ALDH2. In addition, Among the patients with p16-negative OPC, prognosis of the patients with heterozygous ALDH2 (ALDH2*1/2*2) tended to be poorer than that of the patients with homozygous ALDH2 (ALDH2*1/2*1 or ALDH2*2/2*2). Although further study consisting of large number of patients is required to draw a definitive conclusion, this finding suggests the possibility ALDH2 polymorphism may be a prognostic factor for the patients with HPV-negative OPC.

The present study established that SPPEC were significantly more common in patients with p16-negative OPC and heterozygous ALDH2. In addition, prognosis of the patients with SPPEC was significantly poorer than of those without SPPEC. These findings constitute one of the possible explanation for the significantly poorer prognosis of the patients with p16-negative OPC and heterozygous ALDH2. Although it is well known that patients with HNSCC frequently tend to have SPPEC, our results suggest that special attention should be paid during follow-up to patients with p16-negative OPC and heterozygous ALDH2 [18].

The disease-specific survival rate of the patients with p16-negative OPC and heterozygous ALDH2 was also lower not only than that of the patients with p16-positive OPC but also than that of the patients with HPV-negative and homozygous ALDH2. While statistical analysis findings did not reach significance, these results suggest that there must be other unknown reasons other than SPPEC for the poor prognosis of the patients with heterozygous ALDH2. One possible explanation is that patients with heterozygous ALDH2 might continue to drink alcohol at a higher level even after the treatment of oropharyngeal cancer [19]. Alcohol drinking after treatment may induce activation of several transcription factors, such as NF-κB, which have been associated with survival, proliferation, invasion, angiogenesis and metastasis of microscopically residual cancers. Another possible mechanism is that OPC of the patients with heterozygous ALDH2 may be more likely to have TP53 mutation and DNA methylations which are associated poor prognosis, due to alcohol consumption [20].

One of the limitations of this study is the use of DNA extracted from paraffin-embedded biopsies or surgical specimens since errors may have occurred due to deterioration of the extracted DNA. The small number of cases in this study is another important limitation. Further multi-institutional studies consisting of a large number of patients using DNA extracted directly from patients’ blood should therefore be performed to further explore the prognostic significance of ALDH2 polymorphism for patients with OPC.

## Conclusions

Smoking habit was found not to be a significant prognostic factor for Japanese patients with OPC. Instead, ALDH2 polymorphism was found to be possible prognostic factor for patients with HPV-negative OPC. The high frequency of SPPEC in the patients with HPV-negative OPC and heterozygous ALDH2 is one of the most likely explanations for this finding, but there must be other unknown reasons for the poor prognosis of these patients. To the best of our knowledge, ours is the first report on the possible impact of ALDH polymorphism and racial difference on the prognosis of patients with OPC.

## Supporting information

S1 Table Individual data of the 82 patients treated at Kobe University Hospital. OS: Overall survival rate, DSS: Disease specific survival rate, F/U period: follow up period, SPPEC: second primary pharyngeal and/or esophageal cancer. ALDH2*1/ALDH2*1 and ALDH2*2/ALDH2*2 are defined as homozygote and ALDH2*1/ALDH2*2 is defined as heterozygote for statistical analysis.
(XLS)

S2 Table Individual data of the 37 patients treated at Tokyo University Hospital. SPPEC: second primary pharyngeal and/or esophageal cancer.
(XLS)
